# Paternal Contributions to Offspring Health: Role of Sperm Small RNAs in Intergenerational Transmission of Epigenetic Information

**DOI:** 10.3389/fcell.2019.00215

**Published:** 2019-10-09

**Authors:** Upasna Sharma

**Affiliations:** Department of Molecular, Cell and Developmental Biology, University of California, Santa Cruz, Santa Cruz, CA, United States

**Keywords:** sperm, RNA, epigenetic, inheritance, embryo development, exosomes, extracellular vesicles, transgeneration effects

## Abstract

The most fundamental process for the perpetuation of a species is the transfer of information from parent to offspring. Although genomic DNA contributes to the majority of the inheritance, it is now clear that epigenetic information −information beyond the underlying DNA sequence − is also passed on to future generations. However, the mechanism and extent of such inheritance are not well-understood. Here, I review some of the concepts, evidence, and mechanisms of intergenerational epigenetic inheritance via sperm small RNAs. Recent studies provide evidence that mature sperm are highly abundant in small non-coding RNAs. These RNAs are modulated by paternal environmental conditions and potentially delivered to the zygote at fertilization, where they can regulate early embryonic development. Intriguingly, sperm small RNA payload undergoes dramatic changes during testicular and post-testicular maturation, making the mature sperm epigenome highly unique and distinct from testicular germ cells. I explore the mechanism of sperm small RNA remodeling during post-testicular maturation in the epididymis, and the potential role of this reprograming in intergenerational epigenetic inheritance.

## Introduction

The possibility that our life experiences can influence phenotypes in our descendants has tremendous implications for basic biology and public health and policy (Jirtle and Skinner, 2007). Indeed, there is mounting evidence from worms to mammals, including humans, that parental environment can influence phenotypes in future generations (Rando, 2012; Heard and Martienssen, 2014; Rando and Simmons, 2015). However, the mechanism of such transgenerational inheritance —sometimes referred to as inheritance of acquired traits, or Lamarckian Inheritance— remains mysterious. The inheritance of acquired traits was previously refuted, as there was no known mechanism for the environment to alter the genetic material (DNA) transmitted from parents to offspring. With advances in the field of epigenetics, the inheritance that is not based on DNA sequence but how the DNA sequence is utilized, there is a renewed interest in transgenerational inheritance. Epigenetic information carriers (unlike DNA) are highly dynamic and are often modulated by environmental conditions (Sharma and Rando, 2017), suggesting that the environment experienced by parents may influence the phenotype of offspring via alterations to the gametic “epigenome.” However, demonstrating intergenerational inheritance has been challenging in mammals, as such inheritance is a mechanistically complex process that would require epigenetic information to be maintained throughout the disruptive process of epigenetic reprograming during gametogenesis, carried in gametes, delivered to embryos at fertilization, and then influence embryonic development. Furthermore, the inheritance of acquired traits has been discredited by many, because of the so-called “Weismann barrier” that prevents transmission of information from somatic cells to germ cells (and thus to offspring). Paternal contributions to intergenerational epigenetic inheritance have been considered especially unlikely, because of the sheer difference in size between sperm and oocytes.

Challenging these preexisting notions about inheritance, many recent studies in mammals provide evidence that paternal exposure to different environmental stressors, such as diet, psychological stress, toxicants, etc., can influence offspring phenotypes, and sperm epigenome plays a pivotal role in the transmission of such phenotypes. Here, I review recent studies on intergenerational inheritance via male germline, discuss the potential role of small RNAs as epigenetic information carriers in sperm, and explore the mechanisms of such inheritance. Intergenerational inheritance involves transmission of parental environmental effects via exposure of the developing embryo and/or germ cells to the environmental insult (transmission from parent to offspring or grandoffspring). For instance, if a female (F0) pregnant with male offspring is exposed, both its embryo (F1) and germ cells of the embryo (F2) are directly exposed. On the other hand, transgenerational inheritance affects generations that were not directly exposed to the environmental insult (F3 and onwards) (Heard and Martienssen, 2014). I primarily discuss examples of intergenerational inheritance as it is more widely studied in mammals and few examples of transgenerational inheritance are discussed. In addition, I mainly focus on rodent studies, the readers are directed to some excellent recent reviews on transgenerational inheritance in various model organisms (Rando, 2012; Heard and Martienssen, 2014; Rechavi and Lev, 2017; Boskovic and Rando, 2018; Perez and Lehner, 2019). Understanding the mechanism of intergenerational inheritance has important implications for the etiology of human diseases. Many common metabolic disorders, such as diabetes, have both genetic components and contributions from a patient’s lifestyle and environment. Only a fraction of the heritability of such diseases can be explained by genetic variation; instead, it is now increasingly appreciated that epigenetic inheritance likely contributes to such conditions (Rando and Simmons, 2015).

## Intergenerational Transmission of Paternal Environmental Effects

Multiple studies in mammals over the past decade have demonstrated that parents pass on information about their environment to their children. In humans, undernutrition in parents has been linked to metabolic diseases in children, with epidemiological studies of the Dutch Hunger Winter of 1944–1945 (Lumey et al., 2007) providing evidence for effects of maternal starvation during pregnancy on the metabolic health of children. In case of the male germline, studies on three generations of family members in Overkalix, Sweden, demonstrated a correlation between food availability to grandfathers and health and mortality rates in their grandchildren (Kaati et al., 2002; Pembrey et al., 2006). Due to the long term interaction of the mother and the developing fetus in the womb, maternal contributions to offspring phenotypes seem plausible. Indeed, alcohol consumption during pregnancy can lead to fetal alcohol syndrome in humans (Jones and Smith, 1973). However, except for a few studies (Huypens et al., 2016), in most of the studies on maternal exposure, it is difficult to separate environmentally induced molecular changes in the oocyte from the direct effect of the environment on the developing fetus (Rando and Simmons, 2015). On the other hand, as fathers mostly contribute just sperm to the developing offspring, mechanistic investigations of paternal effects are more straightforward, and are of great interest (Rando, 2012).

A large number of laboratory studies in rodents have linked paternal treatment regimes with changes in offspring phenotype, such as dietary alterations (Anderson et al., 2006; Carone et al., 2010; Ng et al., 2010; Fullston et al., 2013; Lambrot et al., 2013; Grandjean et al., 2015; Chen et al., 2016; Huypens et al., 2016; Sharma et al., 2016), psychological stress (Dietz et al., 2011; Rodgers et al., 2013; Gapp et al., 2014; Rodgers et al., 2015; Wu et al., 2016), odor fear conditioning (Dias and Ressler, 2014), exposure to endocrine disruptor vinclozolin (Anway et al., 2005) and ethanol exposure (Rompala et al., 2018). In general, to study intergenerational inheritance of paternal phenotypes, male mice are exposed to different environmental conditions (diet, stress, etc.) and mated with control females. The timing and length of exposure vary in different studies and mostly involves either exposure from weaning to sexual maturity or during fetal development. Next, offspring phenotypic changes are monitored, such as altered glucose metabolism in response to a high-fat diet or depressive-like behavior in response to paternal chronic stress.

In rats, paternal consumption of high-fat diet leads to glucose intolerance in F1 female offspring (Ng et al., 2010). Similarly, male mice fed a low protein diet (10 vs. 19% protein, by mass) were found to sire offspring with altered hepatic cholesterol biosynthesis, relative to control offspring (Carone et al., 2010). Additional studies in mice have used interventions ranging from preconception fasting to fetal undernutrition to link paternal nutrition to glucose metabolism in offspring (Anderson et al., 2006; Jimenez-Chillaron et al., 2009). Moreover, multiple studies have investigated the effects of parental psychological stress, such as social defeat stress, early life trauma and chronic variable stress on offspring phenotypes (Dietz et al., 2011; Rodgers et al., 2013; Gapp et al., 2014). These studies report that paternal exposure to psychological stress usually leads to reduced stress sensitivity in offspring, and altered cortisol levels and glucose metabolism often accompany such phenotypes. The earliest studies in transgenerational inheritance examined the effects of exposure of pregnant female rats to endocrine disruptor Vinclozolin. F1 male offspring of vinclozolin exposed mothers displayed infertility, and this defect was transmitted to males until F4 generation (Anway et al., 2005), suggesting that paternal environmental conditions can influence the health of offspring across multiple generations. In recent years, exposure to other toxicants such as carbon tetrachloride, and drugs such as nicotine and cocaine have also been reported to influence offspring phenotypes (Zeybel et al., 2012; Vassoler et al., 2013; Vallaster et al., 2017).

It is still not clear how paternal exposure is linked to phenotypes observed in offspring, and whether those effects are adaptive. In fact, as discussed above, in most cases transmission of paternal environmental effect increases disease susceptibility in offspring. One explanation for such phenotypes comes from the “thrifty phenotype” hypothesis (Hales and Barker, 2001), wherein a compromised *in utero* environment programs offspring for a similar environment after birth, and thus, links poor fetal and infant growth with increased risk of metabolic diseases in adults born in an unmatched *ex utero* environment (Miska and Ferguson-Smith, 2016). For instance, poor nutrient availability during early development programs offspring to withstand a similar environment, leading to diabetes and obesity in subsequent generations in the absence of nutrient deprivation. Furthermore, an adaptive response might only be revealed when offspring are challenged with a specific environmental insult. It was reported that paternal preconception exposure to nicotine induced a higher metabolic tolerance for nicotine as well as other xenobiotics such as cocaine (Vallaster et al., 2017). The offspring showed higher hepatic expression of various genes involved in xenobiotic clearance, suggesting that in the case of paternal exposure to nicotine, the phenotypes observed in offspring are not specific to nicotine but broadly affects offspring tolerance to additional xenobiotics. Interestingly, this protective response was only revealed in offspring that were pre-exposed to nicotine, suggesting that the phenotypes observed in offspring of exposed fathers are influenced by the interaction of paternal environment with the environment of the offspring (Vallaster et al., 2017).

## Transmission of Epigenetic Information via Sperm

Despite the wealth of knowledge that paternal environment can influence offspring health, the mechanism of this inheritance is not well-understood in most cases. The likeliest scenario is that epigenetic information is delivered to the zygote by sperm, although alternative information carriers such as seminal fluid, microbiome, and female’s judgment of male quality can also play a role (Dietz et al., 2011; Rando, 2012). Use of assisted reproduction methods such as *in vitro fertilization* (IVF) and foster mothers, where the only contribution from parents is their gametes, allows a direct test of whether paternal environmental information is passed via sperm. A few recent studies reported the effects of paternal exposure (such as diet, stress, etc.) on offspring generated via IVF, similar to those seen in offspring produced using natural mating, suggesting that paternal environmental information is indeed transmitted by sperm (Dias and Ressler, 2014; Chen et al., 2016; Huypens et al., 2016; Sharma et al., 2016). Overall, these studies suggest that paternal environment can influence the health of offspring and this information is transmitted via sperm epigenome, and raise the crucial questions: (1) which epigenetic information molecules in sperm carry environmental information? (2) how does the environment influence those epigenetic signaling molecules, and (3) how do those signals influence offspring gene expression and development? Recent studies provide key insights into these mechanistic questions about the process of intergenerational epigenetic inheritance, as discussed below.

## Epigenetic Information Carriers in Sperm

Epigenetic inheritance, the inheritance of phenotypic changes in the absence of changes in DNA sequence, is essential for the maintenance of cell states through cell division. In addition to epigenetic inheritance of cell states during organismal development, there is a growing body of evidence that epigenetic information can be transmitted from one generation to the next, with famous examples including RNA interference in *C. elegans* and paramutation in maize (Fire et al., 1998; Alleman et al., 2006; Heard and Martienssen, 2014; Holoch and Moazed, 2015). Studies of cell-state and transgenerational epigenetic inheritance have identified chromatin structure, DNA modifications, small RNAs, and prions as the main molecular carriers of epigenetic information (Sharma and Rando, 2017). The three most well-characterized epigenetic marks in sperm include histone occupancy and histone modifications, cytosine methylation of DNA, and small non-coding RNAs. Here, I focus on the role of small RNAs in intergenerational inheritance, and briefly review the current understanding of the role of chromatin and DNA methylation in such inheritance [for in depth reviews on epigenetic inheritance via chromatin and DNA methylation, please refer to Heard and Martienssen (2014), Miska and Ferguson-Smith (2016), Boskovic and Rando (2018)].

### Chromatin

In eukaryotes, the genomic DNA is wrapped around a core of histone proteins to form a nucleoprotein complex known as chromatin (Kornberg and Lorch, 1999). This wrapping of DNA around the histone core regulates gene expression by modulating the accessibility of the underlying DNA sequence. Furthermore, post-translational modifications of histone proteins add another layer of regulation by recruiting various chromatin modifying enzymes (activators and repressors) to regulate gene expression. Inheritance of chromatin states from one generation to the next in multicellular organisms is complicated by the fact that chromatin undergoes dramatic changes during gametogenesis, fertilization, and early development (Yadav et al., 2018; Hao et al., 2019). In mammals, during the process of spermatogenesis, the majority of canonical histones are replaced with transition proteins, which in turn are replaced with smaller basic proteins known as protamines (Balhorn, 2007). Importantly, a small fraction of the genome escapes this remodeling. The specific regions that maintain histone occupancy during spermatogenesis is unclear, some studies show that a subset of developmentally important gene promoters retain histones (Hammoud et al., 2009, 2014; Brykczynska et al., 2010), while other recent studies report histone retention at repeat-rich regions of the genome (Carone et al., 2014; Yamaguchi et al., 2018). These conflicting results are potentially due to differences in the histone retention assays used in these studies and suggest that potentially multiple populations of nucleosomes exist in mature sperm, and depending on the protocol used, different populations are detected (Rando, 2016). It is unclear whether persisting histones in mature sperm play a role in intergenerational inheritance. A study reported that histone retention did not change in obese and lean men (Donkin et al., 2016). On the other hand, a role for histone modifications in such inheritance is suggested —carbon tetrachloride exposure lead to altered histone acetylation at specific promoters in mouse sperm (Zeybel et al., 2012). Importantly, studies from model organisms and rodents demonstrate that paternal mutations in genes coding for chromatin regulators cause altered phenotypes in offspring that do not inherit the mutation *per se* (Chong et al., 2007; Siklenka et al., 2015). For instance, overexpression of a histone lysine 4 demethylase KDMA1(LSD1) during spermatogenesis lead to impaired development of offspring for three generations, even in offspring lacking KDM1A overexpression (Siklenka et al., 2015). These studies suggest that correct histone modifications and chromatin structure during spermatogenesis is crucial for proper offspring development, and chromatin potentially plays a role in transmission of paternal environmental effects and motivate future studies to elucidate the mechanisms of such inheritance (Rando, 2016).

### DNA Methylation

Methylation on the fifth carbon of cytosine results in the formation of 5-methylcytosine (5mC), which is one of the most well-characterized epigenetic information carriers. For instance, cytosine methylation is required for germline epigenetic inheritance of imprinted genes — genes that are expressed from only the maternal or paternal alleles and thus, retain the “memory” of the gender of germline they developed from Bartolomei and Ferguson-Smith (2011). As with chromatin, DNA methylation is also globally erased and reset during two rounds of reprograming, one in primordial germ cells during gametogenesis and the other in preimplantation embryos shortly after fertilization (Hackett and Surani, 2013). Certain regions of the genome escape this global erasure, including the imprinting control regions and transposons (Messerschmidt et al., 2014), suggesting that 5mC in sperm could potentially act as a carrier of paternal environmental information in offspring. Indeed, studies have examined the effects of paternal high-fat or low protein diet (Carone et al., 2010; Wei et al., 2014; Shea et al., 2015; de Castro Barbosa et al., 2016; Donkin et al., 2016), paternal folate deficient diet (Lambrot et al., 2013; Ly et al., 2017), and undernutrition (Radford et al., 2014; Holland et al., 2016) on sperm DNA methylation. Although in most of these studies, only a fraction of the differentially methylated regions are maintained in the next generation, these studies provide a strong premise to further investigate the role of DNA methylation in intergenerational inheritance.

### Small Non-coding RNAs

Small RNAs play key roles in multiple, well-established epigenetic inheritance paradigms in model organisms, such as RNA interference in *C. elegans* (Fire et al., 1998) and paramutation in maize (Alleman et al., 2006). Since the discovery of micro RNAs in 1993 (Lee et al., 1993), a multitude of different classes of small RNAs (<40 nts) have been discovered which play critical regulatory roles in biology. The well-studied classes of small RNAs include micro RNAs (miRNAs), endogenous silencing RNAs (endo-siRNAs), and the germ-line enriched piwi-interacting RNAs (piRNAs). In addition, recent advances in RNA-sequencing technology has uncovered additional classes of small RNAs derived from the cleavage of tRNAs known as tRNA-derived small RNAs or tRNA fragments (tRFs). Fragmentation products of ribosomal RNAs are also detected in some biological contexts (Lambert et al., 2019); whether they are functional remains to be determined. Small RNAs primarily function in gene regulation by binding to Argonaute (Ago) family proteins, and this Ago-small RNA complex can regulate gene silencing at different levels, such as (1) transcriptional regulation by targeting DNA methylation and repressive chromatin formation at target gene (Volpe et al., 2002; Chan et al., 2004), (2) post-transcriptional regulation by degradation or deadenylation of target RNA, and (3) translational repression (Bartel, 2009). It is important to note that a subset of tRNA-fragments (mainly tRNA-halves, see below) do not work through the Ago-dependent pathways, and are involved in translational repression by directly interacting with translation machinery, polyribosomes, stress granules, etc. (Ivanov et al., 2011, 2014; Goodarzi et al., 2015; Gebetsberger et al., 2017; Keam et al., 2017; Kim et al., 2017; Dou et al., 2019).

Small RNAs can be distinguished based on their biogenesis and biological function, which in turn is often determined by their size and specific sequence characteristics (Ghildiyal and Zamore, 2009; Czech and Hannon, 2011). miRNAs are ∼22 nt small non-coding RNAs processed from precursor RNA molecules. The precursor RNAs are transcribed from either intergenic regions or introns of protein-coding or non-coding RNA genes. miRNAs can also be transcribed as a long transcript from two or more miRNA genes placed adjacent to each other, called clusters, which have similar seed sequence and thus belong to the same family (Ha and Kim, 2014). The precursor transcripts are processed by Ribonuclease III enzyme Drosha to form pre-miRNAs (Lee et al., 2003). Pre-miRNAs are exported to the cytoplasm by Exportin 5 where they are further processed into mature miRNAs by an RNase III endonuclease Dicer and are loaded onto Argonaute (Ago2) to form the miRNA silencing complex (miRISC) (Hutvagner et al., 2001; Lee et al., 2003). miRISC is then recruited to the target mRNAs (mostly at the 3′ UTR in mammals) through partial base-complementarity and represses gene expression by inhibiting translation (Ha and Kim, 2014).

Piwi-interacting RNAs or piRNAs, so called because of their association with PIWI (P-element induced wimpy testis) clade of Argonaute proteins (Aravin et al., 2006; Girard et al., 2006; Grivna et al., 2006; Lau et al., 2006; Watanabe et al., 2006), are highly expressed in germ cells and are required for male fertility —male mice lacking Piwi proteins are sterile (Carmell et al., 2007). In mammals, two major classes of piRNAs have been reported based on their timing of expression and precursor transcripts: (1) pre-pachytene piRNAs: expressed in fetal and newborn mice and are homologous to various retroelements such as LINE elements, and repress transposon expression to maintain germline genomic integrity (Aravin et al., 2007), (2) pachytene piRNAs: derived from intergenic regions, lack repeat sequences, and are proposed to target spermatogenesis-related mRNAs (Li et al., 2013). In flies and vertebrates, piRNAs have preference for 5′ Uracil and 3′ 2′-*O* methylation (Vagin et al., 2006; Kirino and Mourelatos, 2007). Importantly, primary piRNAs can be amplified by producing secondary piRNAs via the so-called ping-pong cycle of amplification (Iwasaki et al., 2015). Mechanistically, piRNAs repress gene expression at both transcriptional level, by promoting *de novo* DNA methylation (Aravin et al., 2008), and post-transcriptionally by cleaving target transposon mRNAs (Reuter et al., 2011).

tRNA-fragments (tRFs) have only recently been studied in depth (Lee and Collins, 2005; Sobala and Hutvagner, 2011; Anderson and Ivanov, 2014; Kumar et al., 2016), and are therefore relatively not well-characterized. tRNAs are well-known for their role in translation as an adaptor molecule for converting information encoded in RNA molecules to peptide chains by delivering amino acids (Hoagland et al., 1958). Small RNAs derived from tRNAs include a diverse variety of RNA species, such as fragments generated by cleavage in anticodon loop known as tRNA halves, or smaller 18–22 nts fragments produced by cleavage in the D or T loops (Keam and Hutvagner, 2015). In addition, tRFs can be derived from 5′ end, 3′ ends, or middle of pre-tRNAs or mature tRNAs (Lee et al., 2009). tRFs were initially thought to be random degradation products of tRNAs. With the advent of sophisticated deep sequencing methods, recent studies revealed that tRFs are generated in a remarkably site-specific manner and are derived from a subset of tRNA isotypes in a given cell type, suggesting that their biogenesis is a highly regulated process (Keam and Hutvagner, 2015). tRNA cleavage has been characterized in several biological contexts, where it is typically induced in response to stress conditions. In budding yeast and *Tetrahymena thermophila*, RNase T2 family endonucleases process tRNAs (Andersen and Collins, 2012), whereas in mammalian cells exposed to stress, the RNase A family member Angiogenin (encoded by *RNase5*) cleaves tRNAs (Fu et al., 2009). tRFs are found in many cell types but are uniquely highly abundant in the germ cells (Peng et al., 2012; Chen et al., 2016; Sharma et al., 2016). For instance, ∼70% of small RNAs in mature mammalian sperm are composed of tRFs (Peng et al., 2012; Sharma et al., 2016). Treatment of sperm small RNAs with T4 polynucleotide kinase (PNK) in the absence of ATP, which removes cyclic phosphates from the 3′ ends of RNA molecules and, thus, allows cloning of such RNAs for sequencing, revealed additional tRFs and suggested that RNaseA and/or RNase T2 family endonucleases are involved in the biogenesis of tRFs in the male reproductive tract (Sharma et al., 2018). Functions of tRFs are not well-understood, mostly due to their very recent discovery. Different tRFs might act through distinct effector pathways, as evidenced by the fact that tRFs have been reported to regulate gene expression at the transcriptional, post-transcriptional, and translational level. Proposed functions for tRFs include RNA metabolism, global (Sobala and Hutvagner, 2013) and transcript-specific translation inhibition (Ivanov et al., 2011; Goodarzi et al., 2015), ribosome biogenesis (Kim et al., 2017), targeted cleavage of 3′ UTRs (Elbarbary et al., 2009), regulation of apoptosis (Saikia et al., 2014), and regulation of retroviral elements (Sharma et al., 2016; Schorn et al., 2017).

Since mammalian sperm shed most of their cytoplasm, including RNAs, during development, they were long believed not to carry functional RNAs. However, studies in human sperm reported that sperm carry various RNA species (Ostermeier et al., 2002) and a subset of these RNAs (such as, sperm specific transcripts of Protamine-2 and Clusterin) are delivered to the oocyte (Ostermeier et al., 2004), suggesting their retention in internal structures of sperm head. With recent advances in RNA sequencing it is clear that sperm carry both long (>200 nts) and small RNAs (<200 nts). The long RNAs include mRNAs, long non-coding RNAs, and circular RNAs, and miRNAs, piRNAs, and fragmentation products of tRNAs and rRNAs constitute the major classes of small RNAs in mature mammalian sperm (Godia et al., 2018). A comprehensive database for sperm RNAs has been generated as SpermBase which curates both long and small RNAs in sperm of various species (Schuster et al., 2016b). By utilizing *in situ* hybridization, quantitative real-time PCR, microarrays, and RNA high-throughput sequencing, studies report that sperm RNAs are localized in specific compartments. Small RNAs such as miRNAs and tRFs have been sequenced in detergent-treated-sperm heads (Yan et al., 2008; Peng et al., 2012), suggesting their presence in sperm nucleus. Some miRNAs and tRFs are also present in sperm tail, and piRNAs are relatively more enriched in sperm tail compared to sperm head (Sharma et al., 2018). piRNA enrichment in tail is consistent with the localization of remnants of chromatoid body, which is involved in piRNA biogenesis, in sperm midpiece during maturation (Meikar et al., 2011; de Mateo and Sassone-Corsi, 2014). On the other hand, majority of long (>200 nt, including mRNAs) RNAs are enriched in the outer membrane of sperm and about one-third of sperm long RNAs are estimated to be present within the nuclear/peri-nuclear theca (Johnson et al., 2015). Moreover, a subset of sperm RNAs are potentially deeply embedded in the nuclear matrix of sperm nucleus, in complex with the nuclear DNA, which might be difficult to isolate during RNA extraction (Lalancette et al., 2008). Together, these studies demonstrate that a subset of sperm RNAs are embedded in the nucleus and can be delivered to the oocyte at fertilization.

The first evidence that sperm RNAs can influence offspring phenotypes comes from paramutation studies in mice, wherein, microinjection of purified sperm RNAs from mutant mice into fertilized wild type oocytes lead to transmission of mutant phenotypes to offspring (Rassoulzadegan et al., 2006; Wagner et al., 2008; Grandjean et al., 2009). While these studies focused on sperm total RNAs, more recent studies are focused on small non-coding RNAs in sperm which have the potential to regulate gene expression in early embryos. Mature mammalian sperm have a distinct payload of small RNAs, comprising chiefly of tRFs (∼70%), followed by miRNAs as the second most abundant small RNAs and low levels of piRNAs (Peng et al., 2012; Sharma et al., 2016; Hutcheon et al., 2017). Interestingly, abundant tRFs have been reported in sperm of humans, mouse, rat, and bull (Schuster et al., 2016b), suggesting sperm-specific abundance of tRFs is a widespread phenomenon. In addition, fragmentation products of ribosomal RNAs are also present in substantial amount in mature sperm (Chu et al., 2017; Sharma et al., 2018). Some studies have also identified endo-siRNAs and mitochondrial genome enriched small RNAs (mitosRNAs) in mammalian germ cells (Song et al., 2011; Schuster et al., 2016b).

Recent studies provide strong evidence that small RNAs are the environmentally-responsive epigenetic marks in sperm (Fullston et al., 2013; Grandjean et al., 2015; Chen et al., 2016; de Castro Barbosa et al., 2016; Sharma et al., 2016; Zhang et al., 2019). Mice consuming a low protein or high-fat diet have altered levels of small RNAs in mature sperm. A low protein diet leads to upregulation of 5′ fragment of multiple isoacceptors of tRNA-Glycine (tRF-GlyGCC, tRF-GlyTCC, and tRF-GlyCCC), tRNA-LysCTT and tRNA-HisGTT and down-regulation of a miRNA Let7c (Sharma et al., 2016), while a high-fat diet results in an overall increase in the levels of tRFs (∼11%) (Chen et al., 2016). In rats, high fat diet consumption also showed changes in specific small RNAs including, piRNAs (piR-025883, piR-015935, piR-036085), miRNAs (Let7c, miR293, miR880) and tRFs (GluCTC) (de Castro Barbosa et al., 2016). tRFs and Let7 miRNA thus seem to be common small RNAs modulated by diet. Mice fed a western diet (high fat and high sugar) also have altered levels of specific miRNAs in their sperm (Grandjean et al., 2015). Moreover, exposure to toxicants, such as vinclozolin and chronic ethanol exposure, affected tRF and miRNA levels (Schuster et al., 2016a; Rompala et al., 2018). Similarly, exposure to psychological stress, such as early life trauma or chronic stress, lead to changes in levels of specific miRNAs (Gapp et al., 2014; Rodgers et al., 2015).

The implication of sperm small RNAs in intergenerational inheritance of paternal environmental conditions was demonstrated by studies using direct microinjections of small RNAs purified from the sperm of exposed males into control zygotes. In one of the first such studies, mice were exposed to maternal separation coupled with unpredictable maternal stress (MSUS) to induce early life stress. The MSUS mice showed depressive-life behavior, and such behavior was transmitted to their offspring. Injection of RNAs purified from sperm of MSUS mice into one-cell control embryos lead to the transmission of a subset of the behavioral and metabolic trait of MSUS mice to their offspring. This study provided the first direct evidence that paternal environmental information is transmitted by sperm RNAs, and identified a specific set of miRNAs that change in MSUS sperm in response to stress (Gapp et al., 2014). Another study examined intergenerational inheritance of paternal chronic stress and identified nine specific miRNAs that were significantly up-regulated in sperm of exposed mice (Rodgers et al., 2013). Injection of these nine miRNAs (in combination) in control zygotes lead to the transmission of paternal behavioral traits to the offspring (Rodgers et al., 2015). Similarly, in the case of high-fat diet paradigm, microinjection of purified tRFs (30–40 nt size RNAs) into control zygotes reproduced metabolic phenotypes in offspring (as seen by natural mating), suggesting that tRFs potentially act as epigenetic information carriers of paternal dietary information (Chen et al., 2016).

Overall, multiple recent studies suggest that various environmental exposures direct distinct small RNA changes in sperm —diet mainly affects tRFs, while psychological stress influences miRNAs. Whether such differences arise from variation in the methods used for sperm small RNA profiling by different groups or distinct environmental insults are signaled to different classes of small RNAs is not clear. Irrespective, these studies provide strong evidence that small RNAs are environmentally-responsive epigenetic molecules in sperm. Moreover, a recent study reported that both small and long RNAs influence paternal exposure phenotypes of early life trauma in offspring, with long and small RNAs regulating different behavioral traits (Gapp et al., 2018). Although the identity of the specific long and small RNAs involved in such inheritance and their mechanism of regulating offspring phenotype remains unknown, these studies suggest that both small and long RNAs potentially play a role in transmitting complex paternal phenotypes to offspring.

Whether one epigenetic mark is more potent at responding to a certain environmental insult is still not clear. Depending on the type of environmental exposure and the developmental timing of exposure, one epigenetic mark might be more responsive compared to the other. For instance, sperm of mice fed a low protein diet from birth to weaning displayed changes in DNA methylation at ribosomal DNA (rDNA) (Holland et al., 2016), however, if mice are challenged with a low protein diet starting at weaning, levels of specific small RNAs change while rDNA methylation remains unaltered (Shea et al., 2015; Sharma et al., 2016). Although sperm epigenome could be modulated by the environment throughout the life of an organism, it is likely more vulnerable during early development, including embryogenesis and primordial germ cell (PGC) development, when rapid cell division and global reprograming take place. Finally, there could be a cross-talk between different epigenetic marks to relay paternal environmental information to offspring.

## Role of Soma-Germline Communication in Shaping Sperm RNA Payload

The ability of environmental conditions, such as diet, to influence phenotypes in future generations requires that environmental exposure induces changes in the epigenome of gametes. The nature and mechanism of shaping and altering the epigenome of mammalian gametes are not well-understood. One possibility is that somatic cells communicate information to the gametes and, thus, to progeny. Although the Weismann barrier has long been thought to be a problem for such communication (Liu, 2011), over the past decade, an increasing number of studies have suggested soma-to-germline shipment of RNAs in various model organisms (Bourc’his and Voinnet, 2010). Studies in model organisms (e.g., ciliates, flies, and plants), suggest that during gametogenesis RNAs produced in somatic support cells are transferred into developing germ cells where they regulate genomic integrity (Bourc’his and Voinnet, 2010; Martinez et al., 2016). In mammals, there is no direct contact between the somatic support cells and mature gametes, as found in some of the organisms mentioned above —germline and vegetative nuclei are present in the same cytoplasm in ciliates and plants— making soma-germline communication challenging in mammals. Nonetheless, recent studies provide evidence of RNA-mediated soma-germline communication in mammals (Sharma et al., 2016, 2018; Morgan et al., 2019; Trigg et al., 2019).

Post-testicular sperm maturation takes place in the epididymis, a long, convoluted tubular organ where sperm enter after exiting the testis. Intriguingly, recent studies report that sperm small RNA payload undergoes dramatic changes during epididymal transit. Deep sequencing of small RNAs from sperm at various stages of development revealed that while testicular sperm populations such as spermatocytes, round spermatids, and mature testicular spermatozoa are highly enriched in piRNAs (Kuramochi-Miyagawa et al., 2004; Aravin et al., 2006, 2007; Li et al., 2013; Sharma et al., 2018), mature sperm from the distal cauda epididymis are chiefly comprised of tRFs (Peng et al., 2012; Sharma et al., 2016, 2018). Moreover, even the proximal caput epididymis sperm are highly abundant in tRFs (Sharma et al., 2016), suggesting that tRFs are specifically gained upon entry in the epididymis. Interestingly, miRNAs also undergo dynamic changes during epididymal transit, wherein miRNAs such as miRNA 17–92 cluster, are present in testicular spermatozoa, lost in proximal caput epididymis sperm and then reappear in mature cauda sperm (Nixon et al., 2015; Sharma et al., 2018). These studies provide evidence of a novel RNA reprograming event during post-testicular maturation in the epididymis, and raise the question of how small RNAs are gained/lost during epididymal transit.

During the process of spermatogenesis, spermatozoa shed most of their cytoplasm as residual body (Sprando and Russell, 1987) and in the cytoplasmic droplet (Bloom and Nicander, 1961), a subset of small RNAs are potentially lost through this mechanism (Sharma et al., 2018). More puzzling is the gain of small RNAs in transcriptionally silent mature sperm, and suggest that small RNA increase during epididymal maturation is likely not mediated by intrinsic pathways. Indeed, recent studies report astonishing observations that small RNAs in mature sperm are shipped from surrounding somatic epididymis epithelial cells (Sharma et al., 2016, 2018). Epididymis epithelium is highly abundant in sperm-specific small RNAs (tRFs and miRNAs) (Sharma et al., 2016), and by spatiotemporal metabolic labeling of RNAs (Gay et al., 2013), it was demonstrated that mature sperm carry RNAs initially synthesized in the somatic epididymis tissue (Sharma et al., 2018). Together, these studies provide the first evidence of RNA-mediated soma-germline communication in mammals and suggest that post-testicular maturation in the epididymis shapes the small RNA payload of mature mammalian sperm. Intriguingly, sperm DNA methylation is also modulated during epididymal maturation —specific genes were unmethylated in the mature testicular spermatozoa, but remethylated during epididymal maturation (Ariel et al., 1994). Whether such changes in DNA methylation occur genome-wide and have functional consequences during early development remains unknown. In addition to epididymis, other somatic accessory cells of the male reproductive tract, such as, Leydig cells and Sertoli cells, which are in close proximity to the developing germ cells and serve important roles in spermatogenesis (Sharpe et al., 1990; Rebourcet et al., 2014), may also play a role in shaping the epigenome of developing germ cells.

What is the mechanism of this soma-germline communication? Sperm acquire a multitude of lipids and proteins during epididymal transit (Gervasi and Visconti, 2017). A subset of these proteins and lipids are delivered from epididymis to sperm via extracellular vesicles (EVs) secreted from epididymis epithelium, known as epididymosomes (Rejraji et al., 2006; Sullivan et al., 2007; Koch et al., 2015; Sullivan, 2015, 2016). Recent studies revealed that epididymosomes are highly abundant in small RNAs and have a similar RNA payload to that of mature sperm. Moreover, *in vitro* reconstitution studies revealed that epididymosomes can deliver small RNAs to relatively “immature” testicular spermatozoa (Reilly et al., 2016; Sharma et al., 2016), suggesting that epididymosome-mediated delivery of small RNAs is one mechanism of RNA-mediated communication between somatic epididymis cells and sperm.

Deep sequencing of small RNA profiles of epididymis and epididymosomes revealed that only a subset of small RNAs present in the epididymal epithelium are present in epididymosomes (Belleannee et al., 2013; Reilly et al., 2016; Schuster et al., 2016b), suggesting that instead of being passively released by epididymal epithelial cells, subpopulations of epididymal small RNAs are selectively packaged into epididymosomes. The mechanistic basis of selective sorting of small RNAs into epididymosomes remains unknown. Mechanistic studies on RNA sorting into EVs secreted from activated T-lymphocytes revealed that these EVs have a specific miRNA profile which differs from parent cells, with sumoylated hnRNP2AB1 protein being responsible for sorting miRNAs containing a specific tetranucleotide motif into EVs (Villarroya-Beltri et al., 2013). Potentially, similar mechanisms involving specific sequence motifs and/or RNA binding proteins facilitate selective sorting of small RNAs into epididymosomes. Moreover, recent studies in exosomes (a class of 100–150 nm EVs) secreted from HEK293T cells revealed an exosome-specific post-transcriptional modification in tRNAs (Shurtleff et al., 2017), suggesting an additional mechanism of selective sorting of RNAs into epididymosomes. Importantly, recent studies provide insights in the process of delivery of epididymosomal protein cargo to sperm (Zhou et al., 2019). Sperm plasma membrane has unusually high abundance of polyunsaturated phospholipids which compartmentalize its proteins and lipids into specific domains known as lipid rafts (Kawano et al., 2011). Studies suggest a role of these lipid rafts in coordination of initial epididymosome-sperm interaction (Girouard et al., 2009). Since epididymosomal membrane-bound proteins are not detected on sperm (Gatti et al., 2005), it is speculated that epididymosomes adhere transiently to sperm by tethering to receptors at the post-acrosomal domain of the sperm head, which is followed by formation of a fusion pore (Nixon et al., 2019; Trigg et al., 2019; Zhou et al., 2019).

Intriguingly, epididymosomes are a heterogeneous mix of EVs that can be classified into subcategories based on their size and biogenesis (Sullivan, 2015). The various subpopulations of epididymosomes potentially target different cell types. For instance, a subset of epididymosomes may communicate with maturing sperm (Reilly et al., 2016; Sharma et al., 2016, 2018), another subset between different regions of the epididymis (e.g., lumicrine signaling) (Belleannee et al., 2013), and a third subset between the epididymis and the oviduct following delivery to the female reproductive tract via the seminal fluid. Moreover, epididymosome-mediated small RNA delivery to sperm could potentially be regulated at various levels, for example, by regulating the ability of different EVs to interact with sperm and, possibly, by regulating the subset of sperm that receive cargo from specific classes of EVs.

Other mechanisms of RNA-mediated soma-germline communication potentially involve direct shipment of RNAs to sperm in a complex with RNA binding proteins (RBPs), as has been seen in other mammalian cells (Wang et al., 2010; Arroyo et al., 2011; Sarkies and Miska, 2014). Finally, since ejaculated sperm still has a substantial distance to travel before fertilizing the oocyte in the female reproductive tract, EVs secreted from prostate gland (prostasomes; Sullivan and Saez, 2013) or the female reproductive tract (Al-Dossary et al., 2013), potentially further alter the small RNA payload of fertilization-competent sperm.

The functional significance of epididymis specific small RNA remodeling during post-testicular maturation is not well-understood. Recent studies report that small RNA shipment from the epididymis to sperm is essential for embryo implantation (Conine et al., 2018). Embryos generated using intracytoplasmic sperm injections (ICSIs) of immature sperm from the proximal caput epididymis exhibited misregulation of multiple regulatory genes throughout preimplantation development and eventually failed soon after implantation (Conine et al., 2018). Remarkably, microinjections of small RNAs (18–40 nt) purified from cauda epididymosomes completely rescued the post-implantation embryonic lethality phenotype, suggesting an essential role of epididymosomal RNAs in early embryonic development (Conine et al., 2018). However, these studies contradict prior studies in mice reporting successful generation of offspring from ICSI using caput sperm (Suganuma et al., 2005). As discussed in Conine et al. (2018), these discrepancies could arise from differences in the mouse strain background, age of the male, the timing of embryo implantation and the procedure used for the preparation of sperm heads for ICSI. The latter is of particular importance, since prior studies have documented that epididymosomes interact with sperm in a site specific manner (Griffiths et al., 2008; Zhou et al., 2019), and therefore, inclusion/exclusion of certain parts of the sperm could result in different RNA payload being delivered to the oocyte. Future studies, using mice defective in one or more pathways of epididymosome biogenesis and delivery, will shed more light on the functional consequences of shipment of RNA from the epididymis to sperm and its influence on offspring phenotypes.

## Environmental Signaling to Sperm RNAs

An understanding of how environmental conditions influence epigenetic marks in the gametes is essential to elucidate how paternal environment affects phenotypes in offspring. As discussed above, numerous studies provide evidence that paternal environmental conditions, such as altered diet or stress, influence small RNA levels in sperm, however, the mechanistic understanding of how any of the environmental exposures noted above cause specific changes in sperm small RNA levels is largely lacking. Broadly, paternal environment can affect sperm small RNA levels by (1) regulating the transcription of small RNA precursors, (2) regulating small RNA processing, (3) modulating small RNA decay/stability, (4) influencing sorting of small RNAs into epididymosomes, and (5) affecting delivery of epididymosomal cargo to sperm.

RNA post-transcriptional modifications provide the most compelling potential mechanism of environmental signaling to small RNA levels. RNA modifications can affect RNA base-pairing, the secondary structure of the RNA, and RNA–protein interactions and, thus, add a new layer of transcriptional regulation (Roundtree et al., 2017). Indeed, sperm small RNAs from mice consuming a high-fat diet showed increased levels of 5-methylcytosine (m5C) and *N*-2-methylguanosine (m2G) (Chen et al., 2016) and mice exposed to ethanol were reported to have higher levels of 5′-methylaminomethyl-2-thiouridine (mnm^5^s^2^U) and formylcytidine (f^5^C) (Rompala et al., 2018). Not only do these modifications provide stability to tRFs (Chen et al., 2016), they potentially play a role in intergenerational inheritance of paternal environmental conditions —deletion of a tRNA methyltransferase, Dnmt2, which adds m5C to specific tRNAs, prevents transmission of paternal high-fat phenotype to offspring (Zhang et al., 2018). The modified tRFs potentially remain stable in the oocyte at the time of fertilization and thus allow transmission of paternal epigenetic information. However, in the studies noted above, 30–40 nt small RNA fraction (comprising chiefly of tRFs) were examined to identify modified nucleotides by liquid chromatography with tandem mass spectrometry (LC-MS/MS). It is crucial to identify the precise small RNAs that are modified and the specific location of the modified residue, to better understand the mechanistic basis and functional significance of altered post-transcriptional modifications in response to various environmental stimuli. A combination of mass spectrometry, RNA-sequencing, and affinity pull-down approaches will help resolve these issues. How do changes in environmental conditions lead to changes in tRF modifications? As the levels of tRNA modifications have been shown to respond to nutrient availability (Laxman et al., 2013), dietary changes could affect the levels of critical metabolites, such as *S*-adenosyl methionine (SAM), resulting in global changes in levels of specific tRNA modifications and alterations in tRNA processing and/or stability (Schaefer et al., 2010; Tuorto et al., 2012; Sharma and Rando, 2017). However, how non-metabolism related environmental exposures, such as ethanol, affect tRF modifications remains mysterious.

**FIGURE 1:**
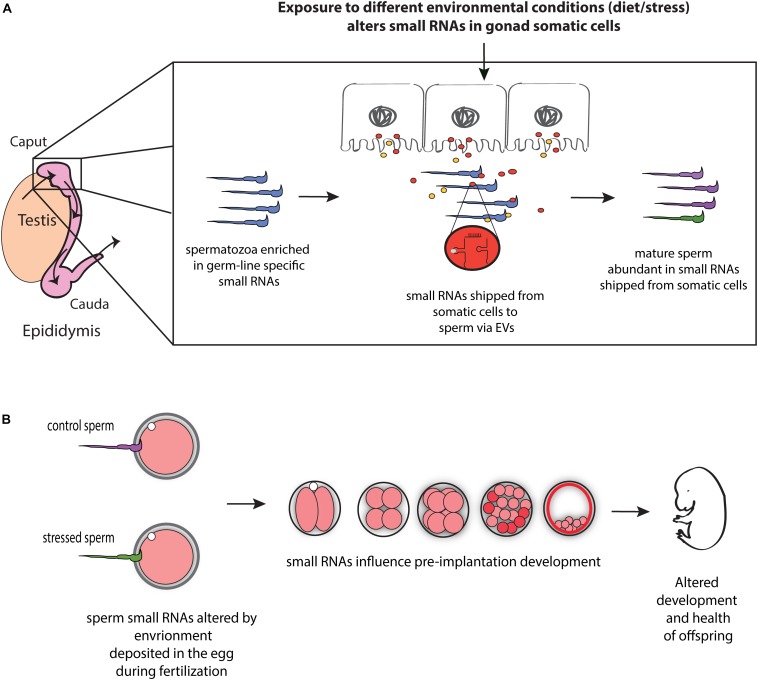
Model for the role of RNA-mediated soma-germline communication in intergenerational inheritance. **(A)** During spermatogenesis and post-testicular maturation, small RNAs generated in the gonad somatic cells (for instance, epididymis) are shipped to sperm via extracellular vesicles (EVs) (and potentially as RNA bound to proteins). The abundance and/or shipment of these small RNAs can be modulated by environmental conditions, leading to an altered small RNA payload of sperm. **(B)** Sperm small RNAs are delivered to the oocyte at fertilization where they potentially affect early embryonic gene expression, resulting in altered early development and health of adult offspring.

Communication between somatic cells and germ cells is not only crucial for the maintenance of germ cell integrity, as suggested in model organisms (Bourc’his and Voinnet, 2010; Martinez et al., 2016), it also provides a potential mechanism for transmitting environmental information to gametes and thereby influencing phenotypes in future generations (Figure 1). For instance, epididymis may serve as an environment sensing organ and transmit this information (in the form of small RNAs) to the maturing sperm, and hence, to offspring. Consistent with such possibility, paternal low protein consumption affects small RNA levels throughout the male reproductive tract, including the epididymis (Sharma et al., 2016). Moreover, paternal exposure to ethanol resulted in similar changes in tRF levels in epididymosomes and sperm, suggesting that paternal environmental information is signaled from the epididymis to sperm via epididymosomes (Rompala et al., 2018). Mechanistically, the authors reported reduced expression of Nsun2 —a 5′ cytosine methyltransferase known to prevent tRF biogenesis by inhibiting cleavage of specific tRNAs (Tuorto et al., 2012)— in the epididymis of mice exposed to ethanol (Rompala et al., 2018), suggesting that reduced expression of Nsun2 in the epididymis leads to increased levels of tRFs in epididymosomes, which in turn affects levels of tRFs in sperm. Overall, these studies suggest that epididymis-mediated remodeling of sperm small RNA payload is modulated by the environment, potentially leading to the transmission of environmental information to the next generation (Figure 1).

## Functional Consequences of Sperm Small RNA Payload

How do changes in sperm small RNAs in response to various environmental conditions result in the transmission of paternally acquired phenotypes in offspring? Mature sperm deliver their RNAs to the oocyte at the time of fertilization, and hence, potentially regulate early embryonic gene expression to affect offspring phenotypes. Indeed, recent studies provide evidence that sperm small RNAs play important, and in some cases, essential role during early development. Microinjection of a synthetic 5′ fragment of tRNA-GlyGCC (tRFGlyGCC) or an inhibitor of tRFGlyGCC −among the most abundant small RNAs in sperm, which is up-regulated in low protein sperm− in control zygotes revealed that tRFGlyGCC represses transcription of a specific set of MERVL retroviral element-driven genes in early embryos (Sharma et al., 2016). While tRFGlyGCC was shown to regulate MERVL expression at the transcriptional level (Sharma et al., 2016), the mechanistic basis of tRF-mediated MERVL-driven gene regulation remains unclear. Intriguingly, genes regulated by tRFGlyGCC were previously shown to be activated during zygotic genome activation in two cell embryos and associated with totipotency program of early embryos (Macfarlan et al., 2012). These findings suggest that tRF-mediated gene expression changes in early embryos potentially lead to altered placental function, which in turn can lead to metabolic phenotypes in offspring as a secondary effect of altered preimplantation development (Sharma et al., 2016). Additionally, 3′ tRFs have been shown to inhibit LTR-retrotransposons in preimplantation trophoblast stem cells (Schorn et al., 2017). Northern blot analysis revealed abundant 3′ tRFs (difficult to sequence with standard small RNA sequencing protocols) in the epididymis and mature sperm (Sharma et al., 2018; Zhang et al., 2018), suggesting that sperm 3′ tRFs potentially play roles in regulating LTR-retrotransposons in preimplantation embryos.

While these studies examined the functions of specific tRFs, another group examined the broad functions of 30–40 nts sperm small RNAs (comprising chiefly of tRFs). Microinjection of 30–40 nts small RNAs purified from sperm of mice fed high-fat diet in control zygotes resulted in altered expression of multiple genes involved in metabolic regulation in eight-cell embryos and blastocysts, suggesting that a cascade of gene expression changes in preimplantation embryos potentially leads to metabolic phenotypes in adult pancreatic islet cells (Chen et al., 2016). Importantly, embryos generated using mice harboring deletion of Dicer, an endonuclease required for miRNAs and endo-siRNAs biogenesis (Ha and Kim, 2014), exhibit reduced developmental potential and altered expression of genes involved in zygotic genome activation (Yuan et al., 2016), further demonstrating that sperm delivered small RNAs play important roles during early development. Overall, these studies point to a role of sperm small RNAs in regulating some crucial steps of early developmental (zygotic genome activation, for instance) and the altered phenotypes in offspring being a secondary downstream effect.

Although, sperm small RNAs are a strong candidate for intergenerational inheritance of paternal environmental effects, many mechanistic questions remain unanswered regarding the delivery and functional consequences of sperm RNAs during early embryonic development. *First*, the RNAs that change in response to environmental conditions are not particularly abundant in sperm. For instance, the most abundant tRFs in sperm are estimated to be at ∼100–100,000 molecules per sperm (Sharma et al., 2016). *Second*, the amount of RNA microinjected in the above-mentioned studies is larger than the amount of RNA a single sperm could deliver, and sperm deliver very low amount of RNA at fertilization compared to the amount of RNA in an oocyte. That said, the microinjection studies, along with studies using genetic manipulation of small RNA biogenesis pathways, provide evidence that altering RNA levels or integrity has profound effects on early embryonic development and health of offspring. Moreover, although sperm small RNAs are present in lower abundance, they likely possess some unique features that make them highly efficient in regulating gene expression —potentially due to post-transcriptional modifications (as discussed above) or pre-loading in some RNA–protein effector complexes. Furthermore, epididymosomes adhering to the surface of the sperm might play important regulatory roles in delivering additional RNAs to the female reproductive tract or the oocyte (Sharma and Rando, 2017; Conine et al., 2018). *Three*, since mammals do not express a known RNA-dependent RNA polymerase —found in various model organisms such as plants and worms, where it is involved in amplifying small RNA signals— it is not clear how sperm small RNAs delivered at fertilization will have prolonged effects on phenotypes in adults. Sperm small RNAs likely regulate some crucial developmental events during the first few cell-divisions, which in turn lead to long-lasting effects on offspring phenotypes. Finally, sperm carry multiple additional non-coding RNAs, such as rRNA fragments, piRNAs, snoRNAs, and long non-coding RNAs, future studies should focus on understanding the roles, if any, of these RNAs in shaping offspring phenotypes.

## Conclusion and Future Perspectives

There is a growing body of evidence, from worms to humans, suggesting that parental environment can influence phenotypes in offspring. However, the mechanistic basis of such inheritance is only starting to be understood. Recent advances in our understanding of intergenerational inheritance revealed that paternal environmental information is transmitted to offspring via sperm and that small RNAs are environmentally-responsive epigenetic molecules in sperm. Intriguingly, epididymis-mediated remodeling of sperm small RNA payload may have consequences for early embryonic development and offspring health. As this remodeling can be modulated by paternal environment, it provides a potential mechanism of transmission of environmental information to the next generation. Future studies will focus on elucidating the mechanism of RNA-mediated soma-germline communication and its consequences on offspring health.

These studies have important implications for human reproductive health. For assisted reproduction in humans, in some cases spermatozoa from testicular biopsies are used for fertilizing oocytes. Therefore, elucidating the functional consequences of sperm small RNA remodeling during post-testicular maturation in the epididymis has important implications for assisted reproduction in humans. Moreover, the seminal fluid also contains a high abundance of exosomes enriched in non-coding RNAs (Vojtech et al., 2014) and EVs are found in most of the bodily fluids such as serum and cerebrospinal fluid (Raposo and Stoorvogel, 2013; Colombo et al., 2014). These observations suggest the intriguing possibility that small RNA loaded EVs might play a crucial role in long-range communication at an organismal level and transmit epigenetic information from distant organs to the developing germ cells, and, hence, to the offspring.

Many important questions remain unanswered regarding the role of sperm small RNAs in intergenerational inheritance. For example, how many pieces of information is packaged in sperm — do sperm carry information about the general quality of life or transmit more specific information about various environmental exposures? How does the environment influence the small RNA payload in sperm? How do changes in sperm small RNA levels affect zygote to give rise to altered phenotypes in offspring? Since different paternal exposures, such a high-fat diet or early life trauma, result in a common metabolic phenotypes (for example, glucose intolerance), are some common pathways targeted by distinct classes of small RNAs? Finally, what is the mechanism of transgenerational inheritance? Since mammals lack a known RNA-dependent RNA polymerase, sperm small RNAs potentially influence early embryonic chromatin and/or DNA methylation states to transmit epigenetic information to the future generations. With recent advances in the field of single-cell omics, a more in-depth examination of sperm and embryonic epigenome will help shed light on these and other outstanding questions, and elucidate the mechanism of transgenerational inheritance of acquired traits.

## Author Contributions

The author confirms being the sole contributor of this work and has approved it for publication.

## Conflict of Interest

The authors declare that the research was conducted in the absence of any commercial or financial relationships that could be construed as a potential conflict of interest.
